# Exploring nationwide patterns of sleep problems from late adolescence to adulthood using machine learning

**DOI:** 10.1126/sciadv.adw1227

**Published:** 2025-09-24

**Authors:** Adrian G. Zucco, Henning Johannes Drews, Jeroen F. Uleman, Samir Bhatt, Naja Hulvej Rod

**Affiliations:** ^1^ Copenhagen Health Complexity Center, Department of Public Health, University of Copenhagen, Copenhagen, Denmark.; ^2^ Section of Epidemiology, Department of Public Health, University of Copenhagen, Copenhagen, Denmark.; ^3^ MRC Centre for Global Infectious Disease Analysis, Department of Infectious Disease Epidemiology, School of Public Health, Faculty of Medicine, Imperial College London, London, UK.

## Abstract

Sleep problems among young adults pose a major public health challenge. Leveraging nationwide health surveys and registers from Denmark, we investigated patterns of sleep problems from late adolescence to adulthood and explored early life-course determinants. We generated life-course embeddings using unsupervised machine learning on data from 2.2 million individuals born from 1980 to 2015. We used this landscape to identify neighboring factors of sleep problems. We observed a substantial increase in self-reported sleep problems among individuals aged 15 to 45, from 34 to 49% between 2010 and 2021, and a 10-fold increase in melatonin use. We also found relevant clusters of sleep-related prescriptions, diagnoses, and procedures with age-specific incidence patterns. Specific childhood adversities, such as sibling psychiatric illness, foster care, and parental divorce, were shared factors across multiple sleep disorders such as insomnia and nightmares. These findings underscore the complex interplay between medical and psychosocial factors in sleep.

## Teaser

Sleep problems from late adolescence to adulthood have increased in the past 10 years and can be linked to complex factors.

## INTRODUCTION

Sleep is essential for physical and mental well-being (*1*, *2*), making the promotion of sleep health an essential focus in public health (*3*). However, understanding sleep is inherently complex. This complexity arises not only due to the biological factors that govern sleep but also due to a multifaceted interplay of environmental, psychological, and sociocultural factors (*4*)—from global warming (*5*) to advancements in technology, such as smartphone usage (*6*). Hence, a complex system approach to the study of sleep is needed to elucidate the patterns, mechanisms, and dynamics that underlie this complex health phenomenon across the life span (*7*).

The transition from adolescence to adulthood is particularly relevant for sleep health. During these formative years, physiological and social vulnerabilities can occur, potentially exerting long-lasting effects on health and life trajectories (*8*, *9*). Furthermore, unique challenges appear later in life, such as work-related stress, caregiving responsibilities, and health issues, which can disrupt sleep (*10*, *11*). Still, fundamental questions concerning sleep problems among younger adults remain unanswered. Before addressing how different factors can lead to sleep problems in young adults, it is important to first “zoom out” to empirically identify sleep patterns and clusters of factors related to these problems. National registers and population-level data present a valuable resource for examining sleep patterns. This process sets the stage for later “zooming in” on the underlying interconnected mechanisms.

The first aim of this study is to evaluate temporal trends in self-reported sleep problems, sleep-related diagnoses, and medication use from late adolescence to adulthood, using nationwide health surveys and registers from Denmark. While previous large-scale studies have provided valuable insights into long-term sleep patterns across broader populations (*12*, *13*), there has been a lack of focus specifically on late adolescence and younger adulthood. Although some cross-sectional studies have explored self-reported sleep problems within this age group (*14*), these efforts have often been limited by small sample sizes (*15*, *16*). In Denmark, nationwide register–based studies have highlighted the comorbidity of diagnosed sleep disorders with depression (*17*), as well as the rising use of the latest sedative medications in the general population and melatonin among young adults (*18*). However, these studies may only capture part of the issue, as many individuals with sleep problems remain untreated and, consequently, unrecorded in registers. To address this limitation, we will complement the register-based data with time trends in self-reported sleep problems from a nationwide survey. This approach will ensure a more comprehensive understanding of sleep patterns at the population level. By combining these data sources, we aim to assess the temporal trends in sleep patterns from late adolescence to adulthood over the past decade.

**Fig. 1.**
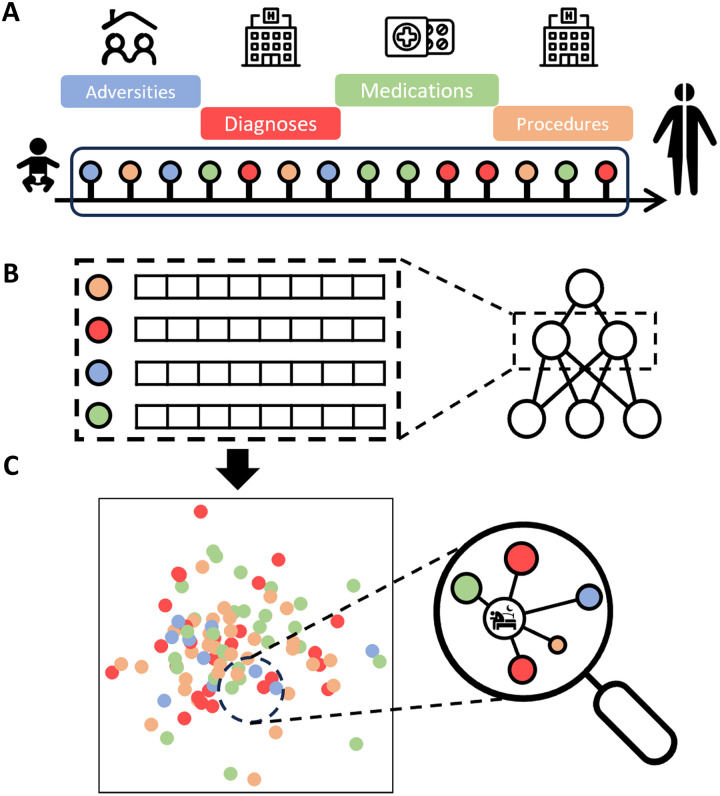
Discovering life-course patterns of sleep problems using machine learning on nationwide registers. (**A**) Life-course data on childhood social adversity, diagnoses, medications, and medical procedures, collected from birth or the start of the corresponding register until a maximum age of 42. (**B**) Life-course embeddings (numerical representations of life-course events) were generated using natural language processing techniques (Word2Vec) by extracting the hidden layer values from the neural network trained. (**C**) Multidimensional embeddings of life-course events were then projected into a two-dimensional landscape and used to explore life-course factors related to sleep problems from late adolescence to adulthood by proximity to known sleep-related diagnoses and medications.

The second aim of our study is to identify life-course factors related to sleep disturbances. To do so, we will use full life-course data in more than 2 million individuals across dimensions of childhood social adversity and medical information such as diagnoses, prescribed medications, and procedures from nationwide registers (*19*). These unique life-course data will be explored by machine learning techniques rooted in natural language processing such as Word2Vec (*20*), which have been successful in selecting disease cohorts by mining electronic health records, learning medical concept embeddings, and encoding clinical histories (*21*–*23*). By learning numerical representations of life-course events, also known as life-course embeddings, we will explore medical and social factors linked to sleep-related conditions (Fig. 1). These life-course embeddings can be interpreted as higher-level abstractions that account for complex dynamics and interactions between multiple factors. This comprehensive mapping of the patterns of sleep problems during the life course is an important step toward enhancing our understanding of sleep disturbances in this population.

## RESULTS

### Increased prevalence of sleep disturbances in late adolescents and adults

**Fig. 2.**
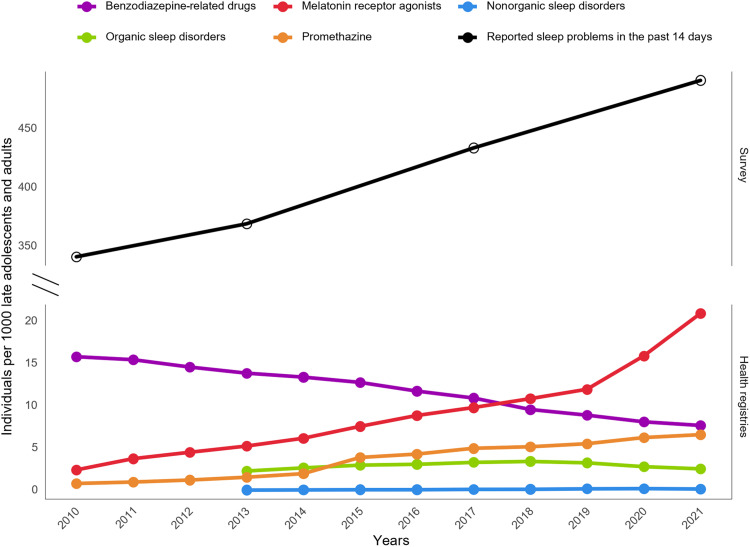
Self-reported sleep problems and prevalence of sleep-related medication and diagnoses in late adolescence and adults from 2010 to 2021. Survey data from the Danish National Health Survey were used to account for self-reported sleep problems collected from individuals aged 16 to 44. Publicly available data from the Danish National Patient Registry and MEDSTAT were used to calculate the prevalence of sleep-related disorders and prescribed sleep-related medication in late adolescents and adults between 15 and 45 years old.

We observe an increase in the prevalence of sleep problems among individuals aged 15 to 45 between 2010 and 2021. This trend is evident across multiple indicators, including self-reported sleep problems, which rose from 34 to 49% over this period. Similarly, there was an increase in the prevalence of melatonin prescriptions (2.43 to 20.9 individuals per 1000 late adolescents and adults per year) and promethazine prescriptions (0.82 to 6.58 individuals per 1000 late adolescents and adults per year) (Fig. 2). In contrast, the use of benzodiazepines decreased in the same period from 15.8 to 7.68 per 1000 late adolescents and adults per year. Diagnoses for organic sleep disorders, such as sleep apnea, remained stable from 2013 to 2021. However, nonorganic sleep disorders such as insomnia increased in the same period from 0.05 to 0.18 individuals per 1000 late adolescents and adults.

### The landscape of sleep problems

**Fig. 3.**
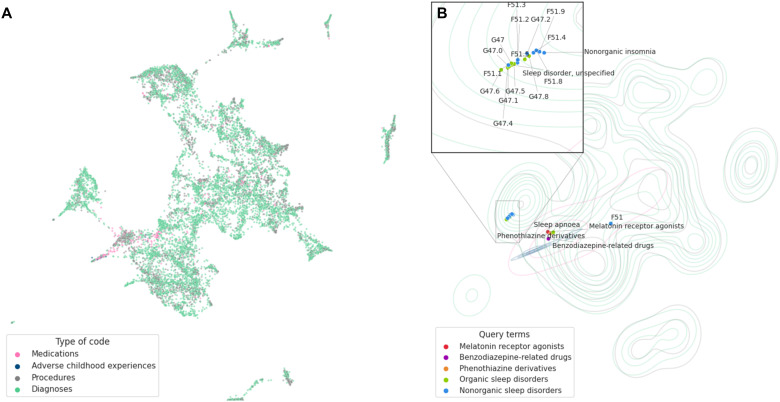
The landscape of sleep-related diagnoses, medications, and medical procedures. (**A**) Dot plot of life-course embeddings for 11,896 terms projected into a two-dimensional landscape using Pairwise Controlled Manifold Approximation (PaCMAP) and colored by type of code. (**B**) Sleep-related terms are highlighted over a kernel density estimation (KDE) of the projected life-course embeddings, showing a colocalization of sleep disorder diagnoses except for the sleep apnea diagnoses, localizing closer to sleep-related prescriptions.

Aiming to uncover the life-course patterns of sleep problems from birth into adulthood and their related factors, we generated representations of life-course events based on natural language processing techniques. By doing so, we encoded multiple life events in the registers (diagnoses, medications, childhood adversity, and medical procedures) into one numerical space, allowing us to explore relationships between life-course factors and clusters of related terms. These multidimensional representations were projected into a two-dimensional landscape, offering a visual representation of the distance between life-course factors and their underlying complexity (Fig. 3A). When highlighting the terms we used as an indication of sleep problems (Table 1), we see that most sleep diagnoses, organic and nonorganic, aggregate together apart from sleep apnea (G47.3), which seems to lie in the proximity of sleep-related medications (Fig. 3B).

**Table 1.** Selected diagnoses and medication indicative of sleep problems.

	Code type	Code
Nonorganic sleep disorders	ICD-10	F51.x
Organic sleep disorders	ICD-10	G47.x
Melatonin receptor agonists	ATC	N05CH
Melatonin	ATC	N05CH01
Benzodiazepine-related drugs	ATC	N05CF
Phenothiazine derivatives	ATC	R06AD
Promethazine	ATC	R06AD02

### Clusters of related medical terms to sleep problems in late adolescents and adults

**Fig. 4.**
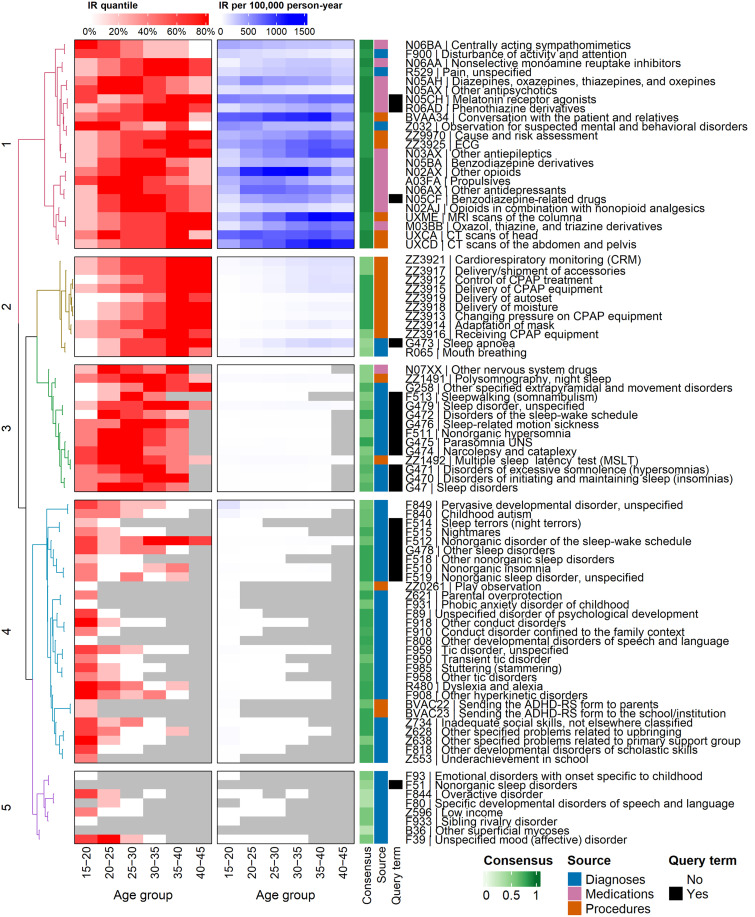
Incidence rates and clusters of sleep-related diagnoses, medications, medical procedures, and their neighboring terms. The dendrogram (left) represents the hierarchical clustering of the 10 closest neighbors to each sleep-related term, highlighting five major clusters of medical terms. IR were calculated for each age group for the period 2010–2021 as cases per 100,000 person-year (blue). IR were divided into five quantiles per medical term and used to categorize age group estimates into each of these quantiles per presented medical code (red). Consensus (green) quantifies the robustness of the clustering approach by representing the frequency of coclustering of each term with other members of its cluster based on different clustering approaches. ECG, electrocardiography; MRI, magnetic resonance imaging; CT scans, computed tomography scans; CPAP, continuous positive airway pressure; UNS, unspecified; ADHD-RS, Attention Deficit/Hyperactive Disorder-Rating Scale.

On the basis of the cosine distance between life-course embeddings, we selected the 10 closest neighbors to sleep-related terms, thereby exploring life-course events related to sleep problems in a young population. Through hierarchical clustering, we identified five relevant clusters (Fig. 4). From the perspective of sleep-related terms, we observe, first, a cluster containing mainly sleep medications (cluster 1). This group presented the highest incidence rates (IR) of all clusters, with the highest IR observed after 20 years of age. Second, a cluster of procedures and diagnoses related to sleep apnea, primarily affecting individuals from age 25 onward (cluster 2). Third, a cluster containing hypersomnias, narcolepsy, parasomnias (including somnambulism), and sleep-related movement disorders [including restless leg syndrome (RLS)] and the related diagnostic procedures polysomnography and multiple sleep latency test (cluster 3). Despite low IR, these medical terms predominantly reflected diagnoses in adulthood. Then, we find a cluster containing sleep terror, nightmare, insomnia, and sleep-wake rhythm disorders (cluster 4). For these diagnoses, we observe the highest IR during childhood and adolescence. The final cluster (cluster 5) included the diagnosis of unspecified nonorganic sleep disorders (F51).

Three clusters captured a more complex picture, linking sleep-related indicators to other diseases, developmental aspects, or social phenomena. The medication cluster (cluster 1) included sleep-related prescriptions (benzodiazepines, melatonin, and phenothiazine derivatives) alongside drugs for pain treatment (e.g., opioids) and mental disorders (e.g., antidepressants and antipsychotics). This cluster also contained imaging procedures such as scans of the head and spine, capturing the interplay between sleep disturbances, mental illness, and pain. Next, cluster 4 including sleep terror, insomnia, and sleep-wake rhythm disorders comprised mainly mental neurodevelopmental diagnoses of childhood and youth, including autism, attention-deficit/hyperactivity disorder (ADHD), tic disorder, hyperkinetic disorder, phobic anxiety, and underachievement in school. This cluster also included nonorganic insomnia (F51.0) and nonorganic disorder of the sleep-wake schedule (F51.2). In contrast to the other diagnoses in this cluster, the highest IR for these sleep disorders were observed at age 25 or higher. The final cluster (cluster 5) with unspecified nonorganic sleep disorders clustered along with indications of low income and mood disorders. This cluster exhibited the lowest IR, reflected in the lack of specificity of its medical terms. We observed that the cluster membership robustness was reduced in terms of the lowest IR.

In sum, our clustering approach shows a distinct pattern of sleep disturbances from late adolescence into adulthood that consist of, on the one hand, sleep-specific clusters (comprising, e.g., sleep apnea–related codes) and, on the other hand, more complex clusters linking sleep to other frequent disorders in the study population such as pain and mental health problems or to psychosocial and developmental problems earlier in life.

### Patterns of childhood social adversity underlying sleep problems

**Fig. 5.**
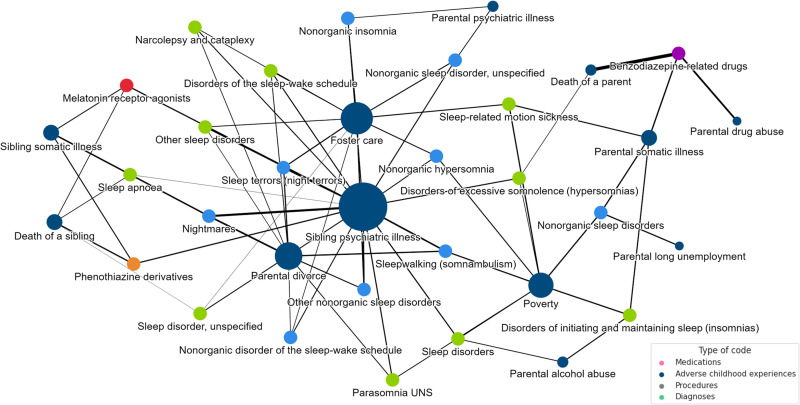
Network of the three closest adverse childhood experiences to sleep-related medical terms. The full network shows the overall complexity through the common shared nodes among adverse childhood experiences, sleep-related medications, and diagnoses. Each node in the network represents a different life-course factor. The size of the nodes indicates the connectedness of each term in the network, and the intensity of the edges reflects the cosine distance between them.

We explored the complexity of early life-course factors potentially related to sleep problems later in life as a network representing the closest neighborhood of sleep-related medications and diagnoses. As most individuals experience at least one childhood adversity, the frequencies of these terms are higher, resulting in lower overall cosine similarity to other terms due to reduced specificity. Hence, we focused on the neighborhood of three childhood social adversities most closely associated with sleep-related terms (Fig. 5).

Our analysis revealed a distinct pattern of childhood adversities underlying sleep problems. Psychiatric illness in a sibling emerged as the most central adversity in the network, followed by placement in foster care and parental divorce. These factors were commonly connected with nonorganic sleep disorders. The strongest relationship in the network, as indicated by the highest cosine similarity, was found between parental death and the prescription of benzodiazepine-related drugs, followed by the association between nightmares and sibling psychiatric illness. Notably, childhood poverty showed a close connection to organic sleep disorders in our network model.

## DISCUSSION

Our study reveals a concerning trend in sleep problems among late adolescents and adults aged 15 to 45 years in Denmark from 2010 to 2021. Using nationwide health surveys and registers, we observed an increase from approximately one-third to almost half of individuals reporting sleep problems over a decade. This increase was also accompanied by a 10-fold increase in melatonin use in the study population. However, this increase in sleep medication partially reflects a shift in prescription practices, with a notable reduction in benzodiazepine use during the same period (*24*). Our findings align with previous analyses in Denmark regarding the growing use of melatonin (*18*) and are consistent with trends observed in other countries, such as the United States, where both reported sleep problems and melatonin use have increased over the past decade (*25*, *26*). Despite these trends, we did not observe a substantial increase in sleep-related diagnoses recorded in hospitals during the same period. This discrepancy reveals a gap between self-reported sleep problems captured in nationwide surveys and the sleep-related medications and diagnoses officially recorded in nationwide registers. The disparity suggests that the increase in sleep problems from late adolescence into adulthood may vary by severity, pointing to a knowledge gap regarding the factors driving this trend. The reported rise in the subjective perception of sleep problems, alongside the growing use of sleep-related prescriptions, warrants further investigation and a more comprehensive approach to addressing sleep problems in young adults.

To map the patterns of sleep problems and related factors, we used machine learning techniques from natural language processing to generate a landscape of life-course factors based on 2.2 million individuals. This landscape allowed us to explore the patterns of sleep-related life-course factors, uncovering relevant clusters of sleep problems, each with characteristic incidence patterns across different age groups. Cluster 1, containing sleep-promoting medications such as melatonin and promethazine, was closely related to mental health medications, including antidepressants and anxiolytics. This cluster presented the highest IR between ages 20 and 40. While previous research has described the comorbidity between sleep issues and other mental health problems based on hospital diagnoses (*17*), our findings suggest that prescription data might reflect this interaction in the broader population and capture treatment by general practitioners. Consequently, prescription data could serve as a valuable proxy for sleep problems. Notably, we did not observe any procedures related to individual psychotherapy in the top 10 closest neighbors of sleep-related terms, despite cognitive behavioral therapy for insomnia being the recommended first-line treatment for insomnia (*27*). This pattern suggests a bias toward pharmacological interventions over psychological treatments. While this gap could be due to the lack of information in our study about psychological interventions outside hospital contacts, it could also indicate a lack of resources for psychological support in the healthcare system or a preference for pharmacological interventions when treating sleep problems. Further studies are needed to explore this aspect.

We observed distinct patterns of IR across the life span, particularly when examining sleep-related diagnoses. Sleep apnea (G47.3), the most prevalent sleep diagnosis, showed higher incidence in the late period of young adulthood and, as expected, clustered with respiratory issues such as mouth breathing (*28*) (cluster 2). Other important sleep disorders, namely, parasomnias (including somnambulism), hypersomnias, narcolepsy, and sleep-related movement disorders, all clustered together (Cluster 3) and showed incidence peaks earlier than sleep apnea. The relationship between these conditions has received limited research attention, although previous studies have shown a higher incidence of RLS in narcolepsy (*29*) and suggested links between RLS and parasomnias. When examining age-related patterns, we found that organic sleep disorders such as narcolepsy, movement-related sleep disorders, and sleep apnea present in clusters 2 and 3 primarily affect individuals in middle to late young adulthood. In contrast, sleep disorders in younger age groups were predominantly linked to emotional regulation difficulties such as nightmares (F51.5) or sleep terrors (F51.4). These early-life sleep diagnoses clustered together with neurodevelopmental disorders such as ADHD, among others, and—interestingly—nonorganic insomnia (F51.0), the prevalence of which peaks in mid-young adulthood (cluster 4). In addition, we observed procedures related to psychosocial assessments and interventions at early ages (cluster 5), suggesting that some of these problems might arise during childhood (*30*).

We further explored whether there were distinct patterns of childhood social adversity occurring before the age of 16 that were related to sleep problems later in life. Previous research has reported associations between adverse childhood experiences and sleep problems in adolescence and adulthood such as insomnia and sleep apnea (*31*, *32*). Our exploration of childhood adversity patterns related to sleep problems revealed that psychiatric illness in a sibling, followed by disruption of family dynamics due to foster care or parental divorce, was the most strongly related factor across multiple indicators of sleep problems. While the causality of these complex, intertwined adversities is not clarified by our analysis, they warrant further investigation. For example, while these social adversities might serve as psychological stressors that trigger mental health problems (*33*), the psychiatric diagnosis of a sibling could also increase the likelihood that individuals in a family are assessed for mental health issues, hence a risk of reporting bias might be present.

We used unique nationwide life-course data to map the landscape in an unselected population with life-course data for more than four decades. In doing so, we expanded on previous work on embedding generation using electronic health records by incorporating information beyond the biomedical field (*21*–*23*), such as childhood social adversities along the full lives of individuals from birth up to 42 years of age. Previous work on comorbidity networks has revealed sex- and age-specific trajectories of organic sleep disorders, showcasing the complexity of sleep disorders and the diversity of long-term effects on health (*34*). The presented Word2Vec model offers a more comprehensive view of the complexity of life-course data compared to traditional comorbidity networks based on pairwise correlations. This is achieved by optimizing the conditional probability of life-course events given the other events in a prespecified window. We propose that encoding contextual information through machine learning approaches could help explore the complexity of life-course data and integrate multiple sources of information from electronic health records to socioeconomic factors available in nationwide registers.

Our selection of Word2Vec for generating global life-course embeddings was informed by recent benchmarks highlighting its strong performance on electronic health record data (*21*–*23*). This relatively simple model facilitated the computation of a comprehensive global embedding space for diverse life-course factors, prioritizing broad life-course co-occurrence over the precise temporal sequence of events. While advanced models based on transformer architectures, such as Life2Vec (*35*), demonstrate superior capabilities in capturing temporal order and achieving high predictive performance, they are based on contextual embeddings. These context-dependent representations can complicate downstream exploratory analyses requiring a unified, global view of the embedding space and may potentially introduce privacy concerns if specific sequences become implicitly encoded (*36*). Notably, our study expands upon previous models trained on electronic health records by incorporating crucial early-life factors, including childhood social adversities and health conditions. This enriched model enabled us to specifically explore the influence of these early experiences on the development of sleep problems later in life, extending from late adolescence into adulthood.

We acknowledge challenges associated with unsupervised learning using epidemiological data, particularly regarding the influence of our modeling choices and the difficulty of defining discrete clusters within a continuous embedding landscape (*37*). To assess robustness, we conducted several validation analyses: we evaluated our Word2Vec embedding stability via data subsampling, we compared it against embeddings from an alternative algorithm (GloVe), and we examined cluster stability through consensus clustering approaches. We observed a moderate correlation (0.56 to 0.60) between cosine distance matrices computed from different data subsets. This result is comparable to the subsampling robustness reported for transformer models (0.66 to 0.67) on nationwide data from Denmark (*35*), suggesting only mild improvements in embedding space stability from more complex architectures. Furthermore, moderate agreement (0.42) between cosine distances from Word2Vec and GloVe embeddings indicates that the model has an impact on the resulting embeddings. Recent approaches unifying embedding spaces could potentially overcome these issues by translating embeddings into universal latent representations (*38*). Regarding cluster stability, consensus clustering indicated that while cluster assignments for frequent life-course factors were robust across different algorithms, they were less stable for rarer factors, such as those present in clusters 4 and 5. Therefore, the presented results must be interpreted within the context of our study population, with particular caution for rarer life-course factors.

Measuring sleep problems and their severity in large populations is also challenging. To address this, we explored the dynamics of sleep problems based on self-reports, prescriptions, and diagnoses from two nationwide data sources, each with its own considerations. Self-reported sleep problems were derived from the Danish National Health Survey (*39*), which has been consistently conducted since 2010 and is weighted to be representative of the Danish population. However, the observed increase in sleep problems is based on subjective reports, primarily reflecting less severe cases. To complement this, we used data on sleep-related medications and diagnoses from nationwide health registers as a proxy for sleep problems. These registers cover the entire population, reducing the risk of selection bias due to Denmark’s universal healthcare system. Nonetheless, these measures are influenced by changes in clinical practices and regulations, such as the observed shift in sedative drug use patterns (*24*). In addition, registered diagnostic data in Denmark are limited to hospital contacts (including inpatient, outpatient, and ambulatory care) and prescriptions, offering only a partial view of general medical practice (*17*). This could have been mitigated by the inclusion of sleep scale measurements at a population level, but comparable sleep scale data were only available for 2021 and are limited to the capital region (*40*). Despite these limitations, our results align with prescription patterns in other countries (*26*) and indirectly with the increased prevalence worldwide in depressive and anxiety disorders (*41*) for which sleep problems are the main symptoms (*42*).

In conclusion, we identified an increasing trend of sleep problems in late adolescents and adults across multiple dimensions, from self-reported to diagnosed sleep disorders. Using a data-driven approach to examine related life-course factors, we uncovered complex patterns of sleep disturbances across various age groups. Our findings highlight the urgent need to address subclinical sleep problems, which affect nearly half of the young adult population in Denmark. We propose that approaching sleep problems as a complex public health issue is essential for developing effective strategies to enhance sleep health and overall well-being in this demographic. Future research should expand on our findings by incorporating additional socioeconomic factors, such as information about labor, education, and adversities occurring after childhood. Furthermore, the integration of diverse data sources with causal analysis could provide a more comprehensive understanding of the underlying complexity driving sleep problems. These pattern recognition approaches in public health could highlight potential leverage points for targeted interventions and policies aimed at improving sleep quality and, consequently, the overall health of young adults.

## MATERIALS AND METHODS

### Public nationwide registries and population-level data

To assess the time trends of self-reported symptoms, diagnoses, and medication related to sleep, we used open-access data on counts and percentages of individuals affected with sleep disturbances. Panel data from a random population sample of more than 150,000 individuals on self-reported sleep problems (ranging from 157,850 to 173,790 individuals across waves) were publicly available from the Danish National Health Survey (*39*) conducted in 2010, 2013, 2017, and 2021. Yearly counts of Danish residents who purchased medications since 2010 were accessible from the Danish online drug use statistics (MEDSTAT) (*43*). Diagnoses from all general and psychiatric hospital contacts since 2013 were obtained from the Danish National Patient Registry (*44*) at www.esundhed.dk/ (more information on Danish health registers available at https://english.sundhedsdatastyrelsen.dk/health-data-and-registers/). Prevalences were calculated using census data on the yearly population of late adolescents and adults, defined as individuals between 15 and 45 years old, provided by Denmark Statistics (https://statistikbanken.dk/; BEFOLK2).

### Life-course data

To explore social and medical factors related to sleep disturbances, we leveraged linked register data from the DANish LIFE course (DANLIFE) cohort (*19*) updated until 31 December 2022. The cohort includes all individuals born in Denmark between 1980 and 2015, corresponding to 2,221,913 people. For this study, we retrieved longitudinal data on diagnoses, prescribed medications, and medical procedures from the birth of individuals or the start of the registers (*19*). Medications were coded following the Anatomical Therapeutic Chemical (ATC) classification up to five characters, medical procedures based on the NOMESCO Classification of Surgical Procedures and the National Health Service’s classification system (SKS) up to six characters, and diagnoses based on the 10th revision of the International Statistical Classification of Diseases (ICD-10) up to four characters. Diagnoses before 1996 were translated from ICD-8 to ICD-10 codes (*45*). Medical data were enriched by social data. By integrating multiple registers and data on siblings and parents, the cohort contains detailed annual information about childhood social adversity across multiple dimensions (*46*). These dimensions involve material deprivation (poverty and parental long-term unemployment), loss or threat of loss (parental or sibling death or life-threatening disease), and familial dysfunction (parental divorce, parental or sibling psychiatric disease, foster care, parental alcohol, or drug abuse). Age-specific IR were calculated for medical terms of interest in the period between 2010 and 2021 based on all individuals present in the DANLIFE cohort at that time, that is, individuals who were alive and had not emigrated. The data processing and summary statistics were performed and visualized in R (*47*).

### Definition of sleep problems

From the survey data, we included individuals who experienced insomnia or sleep problems within the past 14 days of responding to the survey. From nationwide registers, we considered nonorganic and organic sleep disorders recorded at general and psychiatric hospitals including inpatient, outpatient, and ambulatory services (Table 1). We chose three medications as indicative of potential sleep problems. We included benzodiazepine-related drugs (approved for insomnia in the 1980s) and melatonin (introduced in 2007 for insomnia). We also included a phenothiazine derivative, promethazine, due to its off-label use as a sedative despite its origin as a first-generation antihistamine, sold since 2014 under prescription (*24*).

### Representation learning of life-course events

We generate global embeddings for life-course factors by fitting a skip-gram model architecture as originally developed in Word2Vec (*20*). This architecture consists of a three-layer neural network in which the input layer takes a single term representing an event in the sequence of life-course events and the output layer includes all terms included in the model. During training, each life-course event was used as input to predict the surrounding events in the sequence of life-course events of each individual in the DANLIFE cohort. This is done by optimizing a hierarchical softmax function that estimates the conditional probability of each event given its surrounding terms defined by a window parameter. After training, the hidden layer learned a numerical representation, the embedding, of each life-course event. We removed codes with less than 20 observations due to data privacy regulations that prevent us from reporting estimates based on few individuals such as in the case of very rare codes. A total of 11,896 terms were included in the model. Furthermore, on the basis of previous research on representation learning of electronic health records (*21*), distinct codes in the same year were randomly shuffled to avoid bias due to delays in the recording of events in the registers and to preserve the privacy of the individual data. Model specification followed the original Word2Vec implementation (*48*) defined by cross-entropy loss and the Adaptive Gradient Algorithm for optimization. The context of events was delimited to a window size of 100, covering most of the life-course sentence lengths (fig. S1). We trained our model for 25 epochs with a hidden layer size of 200 corresponding to our preferred embedding size and learning rate of 0.05.

The resulting embeddings were projected into two dimensions using Pairwise Controlled Manifold Approximation (PaCMAP) (*49*) based on cosine distance to the 20 nearest neighbors for 450 iterations and default parameters. To facilitate the visualization of the two-dimensional landscape, we applied kernel density estimation (KDE) to the projected embeddings. The model training was performed in Python using Word2Vec from the Gensim library and visualizations using the Seaborn library.

### Unsupervised machine learning and network analysis

We used cosine similarity to quantify the distance between life-course embeddings (equation 1). This metric is commonly used for assessing semantic similarity in word embeddings and is calculated by the dot product of two vectors (*A* and *B*) divided by the product of their magnitudes

cos(A,B)=∑i=1nAiBi∑i=1nAi2·∑i=1nBi2 (1)

Cosine similarity ranges between −1 and 1, with 1 corresponding to full similarity.

To refine our exploration of sleep-related codes, we first defined the relevant search space. We selected the 10 closest neighbors by cosine similarity to each sleep-related term (Table 1), alongside the three most proximate childhood adversities relative to our queried sleep-related terms. We then used two complementary approaches to investigate the relationship between these selected sets of sleep-related codes and childhood adversities.

First, we performed agglomerative hierarchical clustering using the unweighted pair group method with arithmetic mean based on average cosine distances. This method facilitated the aggregation of selected terms into groups of life-course codes based on the assumption that all embeddings are linked through their co-occurrence, thereby allowing exploration of their relative grouping. We partitioned the resulting hierarchical structure into five clusters, determined through a qualitative assessment of the dendrograms. This selection was quantitatively supported by clustering metrics for compactness and separation (*37*), such as the Calinski-Harabasz index, the silhouette score, and the gap statistic, which indicated five as one of the optimal cluster numbers (table S2).

Second, we constructed a network representation to visualize the relationships between the selected childhood adversities and queried sleep-related terms. In this network, the edge thickness represents the cosine similarity between terms, and node size reflects the node degree, illustrating its global connectedness within the network.

### Embeddings and clustering robustness

We assessed the robustness of the generated embeddings and described clusters through several steps. First, to evaluate the stability of the embedding space against data subsampling and initialization randomness, we trained five independent Word2Vec models on distinct data subsets using identical hyperparameters but different random seeds. Spearman correlations between the resulting cosine distance matrices ranged from 0.56 to 0.60. Second, we compared our primary Word2Vec model against an alternative embedding algorithm, GloVe (*50*). The GloVe model was trained using hyperparameters analogous to our Word2Vec model, except for a 0.001 learning rate suitable for its Adam optimizer. The Spearman correlation between the cosine distance matrices from the GloVe embeddings and our reported Word2Vec embeddings was 0.42. Last, to assess the stability of the identified clusters (*k* = 5), we applied several alternative clustering methods to the primary Word2Vec embeddings: hierarchical clustering (single and complete linkage), K-means, and Gaussian mixture models, each constrained to produce five clusters. We then used consensus clustering principles to quantify the frequency with which term pairs co-occurred in the same cluster across these methods, providing a measure of cluster robustness (Fig. 4).

### Proofreading with large language models

Inspired by previous reports on increased equity in scientific writing for nonnative English speakers (*51*, *52*), we also revised parts of the text and codebase using a large language model (Gemini 2.5 Pro). The prompt used was “Proofread, suggest comments and improvements for the following text/code:” All suggestions were carefully reviewed by the authors and adopted only if they improved the clarity and readability of the manuscript and codebase.
