# Supplementary material for: Exploring nationwide patterns of sleep problems from late adolescence to adulthood using machine learning
Source: Sci Adv. 2025 Sep 24;11(39):eadw1227. doi: 10.1126/sciadv.adw1227 (PMC12459426; doi:10.1126/sciadv.adw1227)
Supplement: Supplementary file 1 — Tables S1 to S3 Fig. S1 [file sciadv.adw1227_sm.pdf]

Science Advances
AAAS

Supplementary Materials for

Exploring nationwide patterns of sleep problems from late adolescence to adulthood using machine learning

Adrian G. Zucco *et al.*

Corresponding author: Adrian G. Zucco, adrigabzu@sund.ku.dk

Sci. Adv. **11**, eadw1227 (2025)
DOI: 10.1126/sciadv.adw1227

This PDF file includes:

Tables S1 to S3
Fig. S1

Table S1. Most frequent terms by total counts in life-course sequences.

Code	Counts	Name	Source
J01CE	6268182	Beta-lactamase sensitive penicillins	Medications
G03AA	4666091	Progestogens and estrogens, fixed combinations	Medications
adv9	4093250	Parental long unemployment	Childhood social adversities
adv10	3868152	Poverty	Childhood social adversities
S01AA	3675339	Antibiotics	Medications
J01CA	3286587	Penicillins with extended spectrum	Medications
ZZ0150	2742379	Journal entry	Procedures
Z016	2690374	Radiological examination, not elsewhere classified	Diagnoses
adv8	2469239	Parental divorce	Childhood social adversities
J01FA	2083394	Macrolides	Medications

Table S2. Quantitative assessment of optimal cluster numbers for hierarchical clustering.

	Number of clusters (*k*)								Best *k*
	3	4	5	6	7	8	9	10	
Calinski-Harabasz index	111.38	93.99	**116.28**	96.64	80.77	69.17	75.32	66.98	5
Gap statistic	**-0.65**	-0.92	-0.79	-1.29	-1.77	-1.98	-1.88	-2.05	3
Silhouette score	0.44	0.43	**0.47**	0.45	**0.47**	0.46	0.43	0.42	5, 7

Values of the Calinski-Harabasz index, Gap statistic, and Silhouette score for varying numbers of clusters (k=3 to 10). The "Best k" column indicates the optimal cluster number suggested by the highest value for each metric (in bold).

Table S3. Cluster labels by different clustering algorithms and consensus clustering.

	HC Average Linkage (ref)	*HC Complete linkage*	*HC Single linkage*	*K-means*	*GMM*	*Consensus clustering*
N06BA	1	3	1	2	2	0.898
F900	1	0	1	2	2	0.841
N06AA	1	3	1	2	2	0.898
R529	1	0	1	2	2	0.841
N05AH	1	3	1	2	2	0.898
N05AX	1	3	1	2	2	0.898
N05CH	1	3	1	2	2	0.898
R06AD	1	3	1	2	2	0.898
BVAA34	1	0	1	2	2	0.841
Z032	1	0	1	2	2	0.841
ZZ9970	1	0	1	2	2	0.841
ZZ3925	1	0	1	2	2	0.841
N03AX	1	0	1	2	2	0.841
N05BA	1	3	1	2	2	0.898
N02AX	1	3	1	2	2	0.898
A03FA	1	3	1	2	2	0.898
N06AX	1	3	1	2	2	0.898
N05CF	1	3	1	2	2	0.898
N02AJ	1	3	1	2	2	0.898
UXME	1	0	1	2	2	0.841
M03BB	1	0	1	2	2	0.841
UXCA	1	3	1	2	2	0.898
UXCD	1	3	1	2	2	0.898
ZZ3921	2	4	1	0	0	0.554
ZZ3917	2	4	1	0	0	0.554
ZZ3912	3	0	1	0	0	0.8
ZZ3915	3	0	1	0	0	0.8
ZZ3919	3	0	1	0	0	0.8
ZZ3918	3	0	1	0	0	0.8
ZZ3913	3	0	1	0	0	0.8
ZZ3914	3	0	1	0	0	0.8
ZZ3916	2	4	1	0	0	0.554
G473	2	4	1	2	2	0.446
R065	4	1	1	2	2	0.5
N07XX	4	1	1	1	1	0.5
ZZ1491	3	0	1	1	1	0.5
G258	4	1	1	0	3	0.712
F513	2	1	1	0	3	0.554
G479	4	1	1	0	0	0.635
G472	2	4	1	3	3	0.786
G476	2	4	1	0	0	0.554
F511	2	1	1	0	3	0.554
G475	3	0	1	0	0	0.8
G474	2	4	1	0	0	0.554
ZZ1492	4	1	1	0	0	0.635
G471	2	4	1	0	3	0.705
G470	4	1	1	0	0	0.635

G47	2	4	1	0	3	0.705
F849	3	0	1	3	3	0.6
F840	2	1	1	3	3	0.634
F514	2	1	1	3	3	0.634
F515	4	1	1	3	3	0.75
F512	4	1	1	0	0	0.635
G478	2	4	1	3	3	0.786
F518	2	4	1	3	3	0.786
F510	2	4	1	3	3	0.786
F519	2	4	1	3	3	0.786
ZZ0261	3	0	1	3	3	0.6
Z621	2	4	1	3	3	0.786
F931	3	0	1	3	3	0.6
F89	2	4	1	3	3	0.786
F918	2	4	1	3	3	0.786
F910	4	1	1	3	3	0.75
F808	2	4	1	3	3	0.786
F959	4	1	1	3	3	0.75
F950	2	1	1	3	3	0.634
F985	4	1	1	3	3	0.75
F958	4	1	1	3	3	0.75
R480	2	4	1	3	3	0.786
F908	2	4	1	3	3	0.786
BVAC22	2	1	1	3	3	0.634
BVAC23	2	4	1	3	3	0.786
Z734	2	4	1	3	3	0.786
Z628	2	4	1	3	3	0.786
Z638	5	2	4	3	3	0.571
F818	4	1	1	3	3	0.75
Z553	4	1	1	3	3	0.75
F93	5	2	1	3	3	0.571
F51	2	4	1	4	4	0.446
F844	5	2	3	1	1	0.321
F80	5	2	2	1	1	0.321
Z596	5	2	1	3	3	0.571
F933	5	2	4	3	3	0.571
B36	5	2	0	4	4	0.286
F39	5	2	0	3	3	0.571

Hierarchical clustering (HC) with average, complete and single linkage, K-means and Gaussian Mixture Models (GMM) were computed for a total of 5 clusters (k=5). Clustering algorithms might differ in the cluster labels but represent similar entities. The consensus clustering column quantifies the robustness of the reference clustering approach by representing the frequency of co-clustering of each term with other members of its cluster based on different clustering approaches.

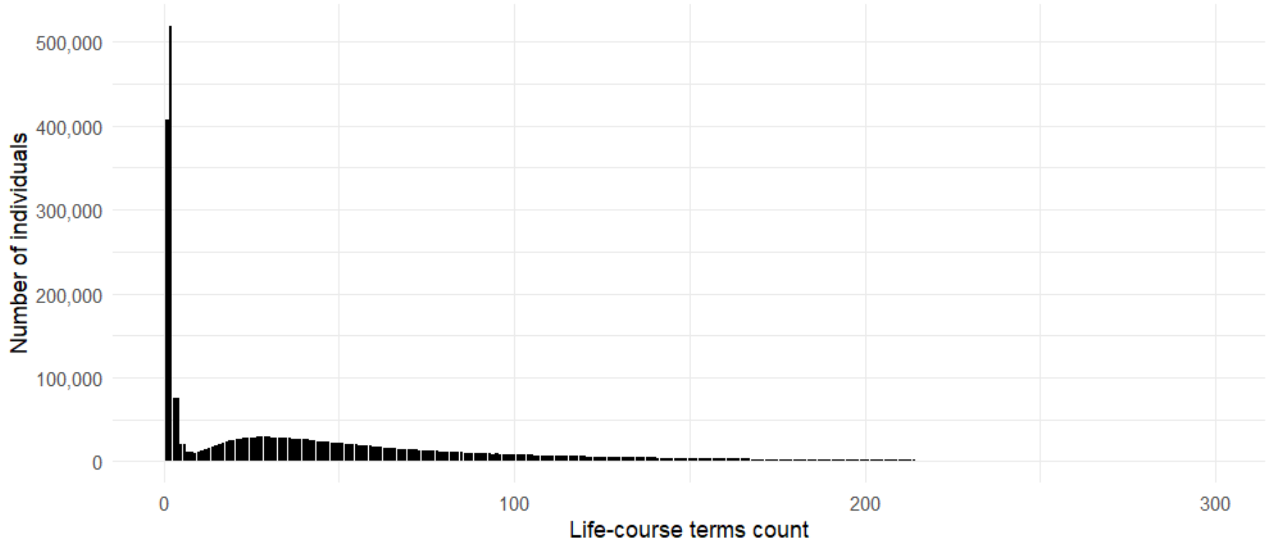

Figure S1. Histogram of life-course term count measured by number of individuals.
Individuals with more than 300 terms in the life course (N = 26832) were excluded from the histogram to facilitate the visualization.
